# The Dual-layer CGuard Stent Is Safe and Effective in Emergent Carotid Artery Stenting and in Tandem Occlusions: a Multi-centric Study

**DOI:** 10.1007/s00062-024-01455-7

**Published:** 2024-09-03

**Authors:** Mousa Zidan, Yves Leonard Voss, Marcel Wolf, Fee Keil, Carolin Brockmann, Christian Gronemann, Nils Christian Lehnen, Daniel Paech, Hannes Nordmeyer, Franziska Dorn

**Affiliations:** 1 Department of Neuroradiology, Bonn University Hospital, Venusberg-Campus1, Gebäude 81, 53127 Bonn, Germany; 2 Radprax MVZ Nordrhein GmbH, Department of Interventional Neuroradiology, St. Lukas-Klinik, Solingen, Germany; 3 Department of Neuroradiology, Mainz University Hospital, Mainz, Germany; 4 Department of Neuroradiology, Frankfurt University Hospital, Frankfurt, Germany; 5 Department of Radiology, German Cancer Research Centre, Heidelberg, Germany; 6 School of Medicine, Department of Health, Witten/Herdecke University, Witten, Germany; 7 Department of Neuroradiology, Medizinische Klinik und Poliklinik IV, LMU-Klinikum, Universität München, Munich, Bayern Germany

**Keywords:** Carotid artery stenting, Tandem occlusion, Dual-layer stent, Thrombectomy, In-stent occlusion

## Abstract

**Background:**

Dual-layer stents have fallen into disrepute after several studies reported high rates of in-stent occlusions in acute stroke treatments. The CGuard stent is a new-generation hybrid dual-layer stent that has been designed to provide less thrombogenicity and to prevent peri- and postinterventional emboli. The aim of the study is to evaluate the safety and efficacy of the CGuard stent for the acute treatment of occlusion or high-grade stenosis of the extracranial internal carotid artery (ICA) in patients with acute ischemic stroke (AIS) with and without concomitant intracranial large vessel occlusion (LVO).

**Methods:**

All patients who underwent emergent carotid artery stenting (CAS) with the CGuard stent were identified and analyzed from the stroke registries from four tertiary German stroke centers. Clinical, procedural, and imaging data were evaluated. Stent patency within 72 h, intracranial hemorrhage, and modified Rankin score (mRS) at discharge were the safety and efficacy end points.

**Results:**

Overall, ninety-six patients were included (mean age 70.2 ± 11.8, 66 males (68.8%), median NIHSS score at admission 11 (7–17), IV lysis: *n* = 44 (45.8%)). Stent placement was successful in all patients. Eighty-three (86.4%) patients had tandem occlusions. In-stent occlusion occurred in 5 patients (5.2%) and 3 patients developed early in-stent stenosis (3.1%). Median mRS at discharge was 2 (1–4).

**Conclusion:**

In this multicenter study, the use of the dual-layer CGuard stent for emergent CAS, particularly in tandem occlusions, was safe and resulted in low rates of in-stent occlusions.

## Introduction

Up to 38% of acute ischemic strokes (AIS) are caused by intracranial large vessel occlusion (LVO) [1]. Endovascular treatment (EVT) has become the cornerstone of treatment, with evidence well established after the publication of positive results from a meta-analysis of five randomized trials in 2015 [2]. Up to 15% of AIS patients present with an extracranial internal carotid artery (ICA) occlusion or high-grade stenosis with a concurrent intracranial thrombembolism, so-called tandem occlusions (TO) [3–5]. These patients require recanalization of the extracranial ICA in order to access the intracranial target lesion for mechanical thrombectomy (MT). Carotid artery stenting (CAS) is performed prior or preferably after [6, 7] after the intracranial procedure in order to maintain the extracranial ICA recanalization. Similarly, in acutely symptomatic thrombogenic sub-occlusive lesions of the extracranial ICA, stenting is frequently performed to cover the culprit lesion to prevent recurring thromboembolism to the intracranial circulation. Finally, stenting may be performed for acutely symptomatic extracranial non-tandem ICA occlusions [8]. There is an ongoing debate about the optimal stent type and medication to be used in these patients.

The additional extracranial procedure adds technical complexity to the treatment and all currently available carotid stents require some form of peri- and postprocedural antiplatelet medication to maintain acceptable rates of thrombotic in-stent occlusions. This raises the question about the risk of stroke-related intracranial hemorrhage [9–11] as well as the rate of in-stent occlusions related to the chosen medication [12]. The CAS procedure further poses a risk of peri- and/or post-procedural embolic events that may negatively influence the outcome of patients after ICA stenting in the acute setting [13]. Embolic events can be attributed to debris dislodgement during stent placement or protrusion of thrombogenic plaque between the stent struts and are especially observed in low metal coverage and open-cell carotid stents [14]. The larger the cell area, the higher the reported risk for post-procedural ischemic events [15]. Accordingly, the carotid stent design is deemed a predictive factor of adverse patient outcomes [15, 16].

Dual-layer stent (DLS) designs have been developed to reduce procedure-related emboli. However, the results from multiple studies in which DLSs were used in the acute setting reported high rates of acute in-stent-occlusions and thrombosis [17–19]. These investigations included either a small number of patients [18, 20], or incorporated insufficient and heterogeneous peri- and postprocedural antiplatelet and anticoagulation therapy [17, 18]. This has deterred neurointerventionalists from using DLSs especially in acute stroke patients with TO [20].

The CGuard stent (InspireMD Inc., Tel Aviv, Israel) is a new-generation double-layer stent. It consists of an inner layer of open-cell nitinol mesh and an outer layer of closed-cell polyethylene terephthalate (PET) mesh designed to trap potential emboli [21, 22]. It has shown promising results in several trials [23, 24], the latest of which included a prospective multi-centric study with a 1-year follow-up that showed a low rate of neurological adverse events in elective patients [25]. However, there is little data on the safety of the CGuard stent in an emergent setting [21, 22]. This study aims to evaluate the safety and efficacy of the CGuard stent for the treatment of acute stroke patients in a retrospective multicenter study.

**Fig. 1:**
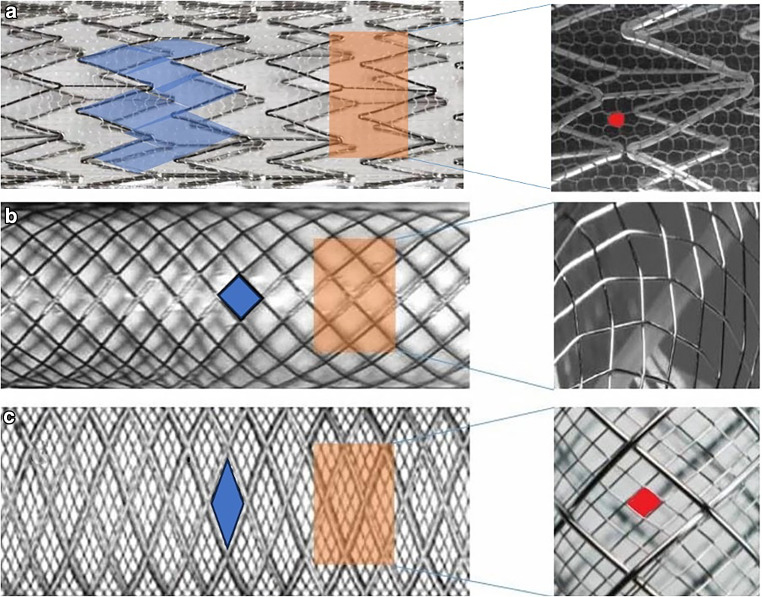
Illustration demonstrating the difference between the dual-layer CGuard stent (**a**) [23], single-layer closed Carotid Wall-stent (Boston Scientific, Santa Clara, CA, USA) (**b**) [35] and dual-layer closed-cell Casper-RX stent (Microvention, Tustin, CA, USA) (**c**) [36]. The free cell size area (blue emphasized space); CGuard: 16.25 mm^2^, Wall-stent: 1.09 mm^2^ and CASPER-stent: 0.38 mm^2^. The pore size (red emphasized space) in CGuard 165 μm and Casper-stent 375 μm

Figure 1 illustrates the design differences between CGuard^TM^ and other commonly used Carotid-stents.

## Methodology

We identified all consecutive AIS patients who underwent treatment with the CGuard stent for an acute symptomatic extracranial ICA occlusion or high-grade stenosis with or without concomitant LVO from the stroke databases from four German comprehensive stroke centers between December 2018 and May 2023.

### Inclusion criteria:

- Patients with a significant neurological deficit with a National Institute of Health Stroke Scale score (NIHSS) at admission of ≥ 4 and a modified Rankin Scale (mRS) score ≥3.
- Available follow-up CT-/MRI imaging of the brain and the cervical arteries including CT-/MR-angiography or Doppler sonography to evaluate stent patency, to detect intracranial hemorrhage and infarction within 72 after the intervention.

### Exclusion criteria:

- Intracranial hemorrhage detected on baseline CT or MRI.
- Simultaneous implantation of another carotid stent of different design.

### Endovascular Treatment and Peri- and Postinterventional Medication

When indicated, intravenous r‑tPA (recombinant tissue-type plasminogen activator) was administrated to eligible patients prior to the endovascular therapy. All acute procedures were performed under general anesthesia (GA) via a transfemoral (TF) approach. A short 8F-sheath was used and an 8F guiding catheter was placed in the proximal common carotid artery (CCA) with support of a 5F selective catheter. In most cases, the stenosis or occlusion of the ICA was passed with a 0.014″ microwire (Traxcess, Microvention, Aliso Viejo, CA, USA or Synchro Standard, Stryker). In cases where intracranial MT was necessary, the use of stent-retrievers or aspiration catheters was at the discretion of the treating neurointerventionalist. A retrograde approach (intracranial procedure before the CAS) was preferred and an antegrade approach was only chosen if the passage of the proximal occlusion was not possible otherwise.

The indication for carotid artery stenting (with percutaneous transluminal angioplasty (PTA) as necessary) was given if a high-grade and hemodynamic stenosis remained after recanalizing the vessel, or if the risk of rapid re-occlusion was considered high due to the configuration of the lesion. In addition, there were no constraints regarding the succession of CAS with or without PTA. Principally, all patients received initially intravenous heparin 3000 UI, and an additional 1000 IU for every additional hour during the intervention.

All patients received intravenous (i.v.) acetylsalicylic acid (ASA) (500 mg) or i.v. weight-adapted Tirofiban before stent-implantation. Technical success was reported when delivery and deployment of the CGuard stent was possible, and no residual occlusion was present. Residual stenosis was identified as stenosis ≥50% as assessed by intra-procedural angiography.

Per protocol after achieving intracranial recanalization and treating the culprit ICA lesion, an angiography of the intracranial circulation is performed to ensure that no new embolization to the intracranial circulation has occurred during the CAS.

Within 24 h after thrombectomy and CAS, patients underwent follow-up imaging with cranial CT or MRI to assess intracranial hemorrhage and infarct size.

The regimen of the postinterventional medication was upon the physician’s discretion. If no contraindications were detected, dual-antiplatelet therapy (DAPT) was started. Platelet function test was performed to identify partial/non-responders.

### Outcome Evaluation

NIHSS and mRS at admission and discharge were evaluated by a neurologist. In case of intracranial hemorrhage (ICH) the assessment criteria were in accordance with the European Cooperative Acute Stroke Study ECASS II [26].

## Statistical Analysis

Baseline patient characteristics were analyzed using descriptive statistics. We performed chi-squared tests for categorical variables and two-sided t‑tests for continuous variables. All calculations were performed using SPSS software (Version 24; SPSS Inc, Chicago, IL, USA). *P* values ≤0.05 were regarded as statistically significant.

## Results

Six patients were excluded from the analysis: Two patients were treated with an overlapping open-cell single-layer stent (Acculink, Abbott Vascular), one patient was treated with an overlapping dual-layer stent (Casper-RX, Microvention) and one patient was treated with an overlapping Carotid Wall Stent (CWS) (Boston Scientific) prior to the CGuard stent in the respective procedures. One patient was initially treated with a CWS for acutely occlusive ICA dissection and the CGuard-stent was later in that procedure deployed as a rescue treatment upon acute thrombotic occlusion of the previously implanted CWS; one patient needed acute treatment for an intracranial atherosclerotic lesion with an intracranial stent in the M1-segment in addition to the CGuard stent for the extracranial ICA.

Overall, ninety-six patients were included: Atherosclerotic extracranial ICA lesions and acute extracranial ICA dissections accounted for 90 (93.75%) and 6 (6.25%) patients respectively. Eighty-three of the patients were treated for tandem lesions (86.4%). The other 13 patients (13.6%) were acutely treated for isolated non-tandem ICA occlusions or sub-occlusive high grade extracranial ICA stenoses without an intracranial occlusion. Complete occlusion of the cervical ICA was observed in 73 of all 96 patients (76%). Mean age was (70.2 ***±*** 11.8) with female patients accounting for 31.2%. Baseline median NIHSS at admission was 11 (IQR: 7–17) and 45.8% of patients received IV thrombolysis. Baseline, demographic, interventional and imaging characteristics of the overall patient population are summarized in Table 1.

**Table 1:** Clinical, radiographic and preprocedural characteristics of the patients

	Acute carotid artery stenting with CGuard (n = 96)
	(Mean ± STD) [N], % (n/N) or median (IQR)
*Age*	70.2 ± 11.8
*Gender*	
Male	68.8% (66/96)
Female	31.2% (30/96)
*Comorbidities*	
Hypertension	57.3% (55/96)
Diabetes mellitus	27.1% (26/96)
Dyslipidemia	63.5% (61/96)
Atrial fibrillation	20.8% (20/96)
Previous cardiovascular disease	36.5% (35/96)
*Preprocedural Characteristics*	
Baseline ASPECTS	8 (7–9)
Baseline NIHSS	11 (7–17)
IV t‑PA use	45.8% (44/96)
Admission mRS	4 (3–5)
Complete occlusion of the cervical ICA	76% (73/96)
*Tandem lesions and intracranial occlusion site*	86.4% (83/96)
Petrous or cavernous segment of ICA	4.8% (4/83)
Terminal segment of ICA	13.2% (11/83)
M1	59.0% (49/83)
M2	18.1% (15/83)
ACA	3.6% (3/83)
PCA	1.2% (1/83)
ICA Dissections	6.3% (6/96)

*NIHSS* National Institute of Health Stroke score, *ASPECTS* Alberta Stroke Program Early CT Score, *IV-t-PA* intravenous tissue plasminogen activator, *ICA* internal carotid artery, *ACA* anterior cerebral artery, *PCA* posterior cerebral artery

All procedures were technically successful. PTA was necessary prior to stent placement in 35.4% of patients (*n* = 34) and was performed after stent placement in 60.4% (*n* = 58) of patients. During the procedures, 93 patients (96.9%) received a single antiplatelet regime (60.4% with ASA and 36.5% with Tirofiban), 3 patients (3.1%) received a DAPT combining ASA and Tirofiban. All of the included patients received i.v. heparin.

Within 24 h of stent implantation the vast majority of patients (91.6%) were treated with DAPT after follow-up imaging ruling out significant intracranial hemorrhage: Fifty-six patients (58.3%) were treated with ASA and Clopidogrel (CPG) and thirty-six patients (37.5%) with ASA and Ticagrelor. Three patients received only ASA as single antiplatelet therapy (SAPT). One patient received SAPT as ASA combined with a direct factor Xa inhibitor for atrial fibrillation. Table 2 shows the different antiplatelet regimens administered.

**Table 2:** Peri- and postprocedural antiplatelet and anticoagulation therapy

	Acute carotid artery stenting with CGuard (n = 96)
	% (n/N)
*Periprocedural antiplatelet and anticoagulation therapy*	
ASA	60.4 (58/96)
Tirofiban	36.5 (35/96)
ASA + Tirofiban	3.1 (3/96)
*Postprocedural antiplatelet and anticoagulation therapy*	
ASA + Clopidogrel	58.3 (56/96)
ASA + Ticagrelor	37.5 (36/96)
ASA + Apixaban	1 (1/96)
Only ASA	3.1 (3/96)

*ASA* acetylsalicylic acid

Intraprocedural acute in-stent thrombus formation occurred during one of the 96 procedures and completely resolved interprocedurally after swift administration of a Glycoprotein IIb/IIIa inhibitor (Tirofiban). Including that case, all stents were patent on the final angiograms and there was no significant residual stenosis upon completion of the interventions in all 96 patients.

CT-angiography or Doppler sonography were performed within 72 h after stenting to assess for in-stent occlusion or re-stenosis. Acute in-stent occlusions occurred in 5 cases (5.2%). One in-stent occlusion occurred on day 3 post stent implantation in a patient treated with acetylsalicylic acid (ASA) as SAPT for more than 24 h; that patient was on oral anticoagulation therapy with Apixaban for atrial fibrillation. Two of the in-stent occlusions occurred in patients treated for dissections. The remaining two cases of in-stent occlusions occurred under routine DAPT.

Three cases of in-stent stenosis (3.1%) were identified on follow-up imaging. One of the cases with in-stent stenosis >70% was re-treated with balloon angioplasty in an elective procedure. The other two cases presented with an in-stent stenosis degree of 50% and no additional treatment was performed.

Median mRS at discharge was 2 (IQR: 1–4). Forty-nine patients (51. %) had a favorable outcome at discharge (mRS <3). Follow-up between 6 to 12-months was available in 52 patients (54.2%) and all stents were patent and no new cases of in-stent stenosis were detected. The formerly described two cases of in-stent stenosis of 50% remained unchanged. These patients underwent Doppler sonography on their long-term follow-up to assess stent patency*.* Table 3 demonstrates the interventional data of EVT and associated complications.

**Table 3:** Periprocedural and interventional characteristics and complications

	Acute carotid artery stenting with CGuard (n = 96)
	(Mean ± STD) [N], % (n/N) or median (IQR)
*Balloon Angioplasty*	
Predilation	35.4% (34/96)
Postdilation	60.4% (58/96)
*Applied stent*	
Stent diameter (mm)	8 (8–9.5)
Stent length (mm)	40 (40–40)
*TICI*	
2a	2.1% (2/96)
2b	52.1% (50/96)
3	45.8% (44/96)
*Stent patency on follow-up imaging in 72* *h*	
In-stent occlusion	5.2% (5/96)
In-stent stenosis	3.1% (3/96)
*Hemorrhagic transformation or intracranial hemorrhage*	
Total hemorrhage of any ECASS type	17.7% (17/96)
HI1	9.4% (9/96)
HI2	2.1% (2/96)
PH1	3.1% (3/96)
PH2	3.1% (3/96)
sICH	5.2% (5/96)
*Clinical outcome*	
mRS at discharge	2 (1–4)

*TICIs* thrombolysis in cerebral infarction scale, *mRS* modified Rankin Scale, *sICH* symptomatic intracranial hemorrhage, *HI* hemorrhagic infarction, *PH* parenchymal hematoma, *ECASS* European Cooperative Acute Stroke Study

There were two cases of in-stent occlusions in each of both DAPT regimens, so no significant association between the type of antiplatelet regimen and in-stent occlusions was observed (chi-square *p* = 0.588). Platelet inhibition testing did not recognize non-response or impaired response to CPG among the five patients with in-stent occlusions.

Among the 5 patients with in-stent occlusions; 1 died due to respiratory failure, 1 didn’t survive intracranial hemorrhage PH2, and 3 patients developed an advanced infarct demarcation of the MCA territory where no further interventions were warranted.

Any intracranial hemorrhage or hemorrhagic transformation was reported in 17/96 patients (17.7%). According to ECASS II classification: there was HI1 class hemorrhage in nine patients (9.4%), class HI2 in two patients (2.1%), class PH1 and PH2 hemorrhage in three patients respectively (3.1 and 3.1%). Two of the PH2 class and two of the PH1 class hemorrhages were associated with neurologic deterioration, one PH2 class hemorrhage was associated with a fatal outcome. Overall symptomatic intracranial hemorrhage (sICH) occurred in five patients (5.2%). There was no significant association between the different antiplatelet regimens and intracranial hemorrhage (chi-square *p* = 0.189).

## Discussion

In our multicentric retrospective analysis of emergent ICA stenting in acute stroke cases the CGuard stent system provided a high technical success rate and comparatively low rates of early in-stent occlusions and intracranial hemorrhage.

We observed intraprocedural thrombus formation in one patient and early in-stent occlusions within 72 h of stent placement in five (5.2%) of our 96 cases. We consider this as evidence of the favorable safety of the CGuard stent compared to the previously reported high occlusion rates for other DLSs. In the literature the early stent occlusion rates reported for the Casper-RX stent were 45% in a series by Yilmaz et al. and 52.4% in a series by Bartolini et al. [18, 20]. Another multicentric study by Pfaff et al. [17] reported intraprocedural thrombus formation in 25 of 160 patients (15.6%) and early in-stent occlusions of Casper-RX stents intraprocedural or within 72 h in 12/160 patients (7.5%).

Currently there is little literature to compare with our observed performance of the CGuard stent for acute stroke cases. Klail et al. reported an acute occlusion rate of 9% after acute stenting of the ICA with CGuard stent in thirty-three cases [22], compared to a rate of 5.2% in our series of 96 patients. Notably, two out of the five in-stent occlusions in our analysis occurred in patients with an underlying dissection, while only 6 of 96 analyzed patients were treated for underlying dissections. Acute dissections are at times technically challenging to treat and appear to be associated with unfavorable outcomes and high risk for in-stent thrombosis in tandem lesion patients [12, 27].

The in-stent occlusion rates published in the literature vary substantially between 2 and 20% [12, 28] and appear to depend heavily not only on the stent type used, but also the intraprocedural medication, rate of performed balloon-angioplasties, and patient characteristics like the rate of underlying dissections [12, 29].

In order to properly classify our positive results, it is important to understand that the CGuard design is different from other dual-layer stents. For example, the Casper-RX consists of a braided metal frame which forms the stent frame and an inner layer of a nickel-titanium alloy; this design results in considerably higher metal coverage when compared to the CGuard. In addition, in other DLSs, the mesh layer lies inside the metal stent frame, instead of outside, and thus lacks structural support to stop plaque intrusion. This can potentially explain the previously reported higher rates of in-stent restenosis [30, 31].

Another technical aspect worth mentioning is that navigating and stenting torturous vessels can be problematic in closed-cell stents [32]. In this regard, the open-cell component in CGuard offers advantages in flexibility and conformability with a large free cell area of 16.25 mm^2^ as compared to 1.09 mm^2^ of the CWS. This possibly contributed to the high rate of technical success in our analysis.

Due to the multicentric nature of our study, the peri- and postprocedural medication regimens were heterogeneous. All patients received either ASA (60.4%) or the glycoprotein IIb/IIIa inhibitor Tirofiban (39.6%) intravenously before stent-implantation. Excluding those patients that developed substantial intracranial hemorrhage detected on follow-up CT within 24 h, all other patients (95.8%) consistently received DAPT within 24 h after stent implantation. The combination of ASA and CPG was the most common DAPT regimen used in 58.3% of patients, ASA and Ticagrelor were used in 37.5% of patients.

Each of the postprocedural DAPT regimens in our investigation was associated with two cases of in-stent occlusions, so we did not observe a significant difference in the effectiveness of the chosen periinterventional or postprocedural medication regimes applied in our analysis. One in-stent occlusion occurred in one patient who only received ASA as SAPT in addition to a factor Xa inhibitor for underlying atrial fibrillation for more than 24 h, which is in alignment with previous evidence of higher rates of in-stent thrombosis in tandem occlusions not treated with DAPT within 24 h of stent implantation [29].

In the publications reporting high rates of in-stent occlusions for other DLSs such as the Casper-RX, as cited above, the medication regimes varied: Yilmaz et al. reported for their Casper-RX patients a regimen of SAPT with ASA at stent deployment and overlapping SAPT consisting of Clopidogrel within 24 h [18]. Whereas, in the multicentric investigation by Pfaff et al. the periprocedural medication was heterogenous. The most common administered regimens were Heparin (38.8%), ASA (57.5%) or glycoprotein IIb/IIIa inhibitors (31.3%) during stent deployment. Surprisingly, the latter was significantly more associated with higher rates of acute thrombus formation *p* < 0.001 [17]. Although in that investigation, 83.1% received DAPT as a postprocedural long-term medication, a SAPT in form of ASA was given to 13/160 (8.1%) patients following the procedure. That SAPT group experienced disproportionally high rates of in-stent thrombosis 6/13 patients. A plausible explanation for the occurrence of acute stent occlusions and thrombus formation in the Casper-RX stents may be the increase of thrombogenic material because of the second nitinol micromesh layer in a setting of insufficient preparation with antiplatelet medication.

The early in-stent occlusion rate observed in our analysis of emergent CGuard stenting is comparable to the in-stent occlusion rates previously reported on the mono-layer CWS for TO in comparable patient collectives under single dose ASA i.v. administered intraprocedural for the first 24 h of stent implantation, followed by DAPT after 24 h, at about 5% early in-stent occlusions [33].

We attribute the comparatively low rate of early in-stent occlusions of 5.2% in our analysis not only to the CGuard stent design providing improved plaque coverage while maintaining low thrombogenicity, but also to the effectiveness of a consistent application of periinterventional SAPT prior to stent implantation plus the consistent application of DAPT within 24 h after stent implantation whenever feasible. In our analysis ASA i.v. as well as Tirofiban i.v. appeared to be safe and effective regimes to prepare for implantation of the CGuard stent in emergent CAS and maintain its patency for the first 24 h.

In this investigation, only 52/96 (54.2%) patients received follow-up imaging at 6 months. This is attributed to the high quota of external stroke patients referred to the comprehensive stroke centers for treatment, which are consecutively transferred back to their local hospitals and neurological rehabilitation centers after treatment.

Although patients with tandem occlusions present a challenging case for endovascular stroke treatments due to the less favorable prognosis and increased risk for ICH [11], in this multicentric analysis favorable outcomes (mRS 0–2) were observed in 51% of the patients at discharge.

In our analysis the overall rate of parenchymal hematoma class PH2 per ECASS II definition was 3.1% and the rate of clinically significant hemorrhages (sICH) was 5.2% under the medication explained above, which is comparable to the hemorrhage rates in the literature on emergent CAS for tandem occlusions [7, 29, 33] or non-tandem ICA occlusions [8].

### Limitations

While our study provides the largest sample size of CGuard stent procedures performed in acute stroke patients to date, including 83 tandem occlusion patients, the retrospective and multicenter nature of the study implies a number of limitations in the form of different patient characteristics, reported and unreported interventional technical aspects of the underlying procedures and heterogenous peri- and postinterventional medication regimes possibly influencing patient outcomes as well as the endpoints in-stent occlusion rate and intracranial hemorrhage rate. This reflects a real-world heterogeneity of stroke EVT approaches for TO and non-tandem ICA occlusions in different stroke centers today. Another limitation of our study is that long-term follow-up data was unavailable in almost half of the cases limiting the long-term assessment of stent patency. Lastly, the modalities performed to evaluate stent patency have varying sensitivity, but the availability of follow-up imaging is, at times, limited.

The optimal timing of carotid artery stenting during EVT remains uncertain. Antegrade approach can facilitate access but delays intracranial recanalization and risks stent entanglement. Conversely, retrograde approach allows quicker restoration of blood flow but risks distal embolization.

Immediate CAS during EVT requires dual antiplatelet therapy to prevent stent thrombosis, posing a risk of hemorrhagic transformation and cerebral hyperperfusion syndrome. Studies show varying rates of intracranial hemorrhage, with some indicating higher rates and others, including the TITAN registry [34], suggesting no increased bleeding risk with immediate CAS. There is no consensus on antithrombotic management for carotid stent placement during stroke treatment, leading to varied practices.

Randomized controlled trials (RCTs) could provide clarity on these issues by comparing outcomes of the antegrade and retrograde approaches, evaluating the timing of CAS, and assessing the safety and efficacy of different antithrombotic regimens.

## Conclusion

In this multicenter study, the use of CGuard for emergent carotid artery stenting in acute stroke including tandem occlusions, resulted in considerably lower rates of in-stent occlusions when compared to previous observations of the other designs of dual layer stents. This could be explained by the difference in stent design, insufficient antiplatelet therapy or a combination of both. Our study shows that the CGuard stent provides reduced thrombogenicity under adequate peri- and postprocedural antiplatelet therapy.

## Data Availability

Individual participant data that underlie the results reported in this article (after deidentification) will be available upon reasonable request. These proposals should be directed to the corresponding author.
